# Newly diagnosed multiple sclerosis in a patient with ocular myasthenia gravis: A case report

**DOI:** 10.1097/MD.0000000000028887

**Published:** 2022-02-25

**Authors:** Jeong Bin Bong, Min A. Lee, Hyun Goo Kang

**Affiliations:** a Department of Neurology, Chosun University College of Medicine, Gwangju, South Korea; b Department of Neurology, Jeonbuk National University Medical School and Hospital, Jeonju, South Korea; c Biomedical Research Institute, Jeonbuk National University Medical School and Hospital, 20 Geonji-ro, Deokjin-gu, Jeonju, South Korea.

**Keywords:** multiple sclerosis, myasthenia gravis, optic neuritis

## Abstract

**Rationale:**

Patients with myasthenia gravis may also have comorbid autoimmune diseases. Since both myasthenia gravis and neuromyelitis optica spectrum disease are mediated by antibodies, they are likely to occur together. However, since multiple sclerosis is an autoimmune disease that is not mediated by a specific antibody, it has fewer immune mechanisms in common with myasthenia gravis than neuromyelitis optica spectrum disease. We encountered a case of newly developed multiple sclerosis in a patient with myasthenia gravis.

**Patient concerns:**

A 46-year-old man was diagnosed with ocular myasthenia gravis 6 years ago and had been taking pyridostigmine to control his symptoms.

**Diagnosis:**

The patient developed right optic neuritis, and multiple sclerosis was suspected based on the brain magnetic resonance imaging findings. However, the required diagnostic criteria were not met.

**Interventions:**

Disease-modifying therapy was not initiated, and clinical progression of the disease was monitored.

**Outcomes:**

One year after the onset of optic neuritis, the patient developed myelitis and was diagnosed with multiple sclerosis, prompting treatment with disease-modifying therapy.

**Lessons:**

When optic neuritis occurs in patients with myasthenia gravis, careful evaluation is necessary while considering the possibility that it may be the first symptom of a demyelinating central nervous system disease. Therefore, it is important to conduct shorter-interval monitoring and symptom screening for patients with neurological autoimmune diseases, such as myasthenia gravis, even if multiple sclerosis is not initially suspected, to achieve early detection of multiple sclerosis.

## 1 Introduction

Myasthenia gravis and multiple sclerosis are autoimmune diseases that affect the neuromuscular junctions and central nervous system (CNS), respectively. The main mechanism of myasthenia gravis is antibody-mediated, while that of multiple sclerosis is T cell-mediated. In part, both these diseases are caused by immune dysregulation induced by the numerical, functional, and migratory deficiency of T regulatory cells, which play an important role in immunologic tolerance.^[1]^ Approximately 25% of patients with autoimmune diseases tend to develop more autoimmune diseases.^[2]^ The co-occurrence of myasthenia gravis and demyelinating disorders is more common than expected by chance.^[3]^ We report a patient with myasthenia gravis, an antibody-mediated disease, who developed multiple sclerosis, a nonantibody-mediated disease, several years later.

## 2 Case presentation

A 46-year-old man presented with sudden onset of decreased vision in the right eye that occurred suddenly 2 days prior. Six years ago, the patient had undergone several tests for ptosis and diplopia. A significant decremental response was observed in the repetitive nerve stimulation test performed on the orbicularis oculi muscle. Anti-acetylcholine receptor antibody test result was positive. Consequently, he was diagnosed with ocular myasthenia gravis and was prescribed 180 mg/d of pyridostigmine to be taken orally. The patient had a mean quantitative myasthenia gravis score of 2. Chest computed tomography performed at that time did not reveal any thymic abnormalities. He was able to continue his daily life without much discomfort, despite only taking pyridostigmine with no immunosuppressants. Therefore, he was followed up on an outpatient basis.

During this visit, neurological examination revealed identical pupils with normal pupillary reflexes. However, a relative afferent pupillary defect was observed in the right eye. The visual acuity of the right eye enabled recognition of fingers at a distance of 30 cm from the eye, while that of the left eye was 0.8. Fundus examination revealed swelling of the right optic disc. There were no defects in the extraocular muscle movements, as well as orbital pain. The results of all the other cranial nerve tests were normal. Moreover, there were no motor or sensory deficiencies in the upper and lower extremities.

Blood workup including complete blood count, electrolytes, liver and renal function tests showed normal results. Erythrocyte sedimentation rate, C-reactive protein and fluorescent antinuclear antibody test results were normal as well. The results of the anti-aquaporin 4 antibody, antimyelin oligodendrocyte glycoprotein antibody, and oligoclonal band of cerebrospinal fluid were negative; while the immunoglobulin G index did not show an increase from the baseline of 0.7.

**Figure 1:**
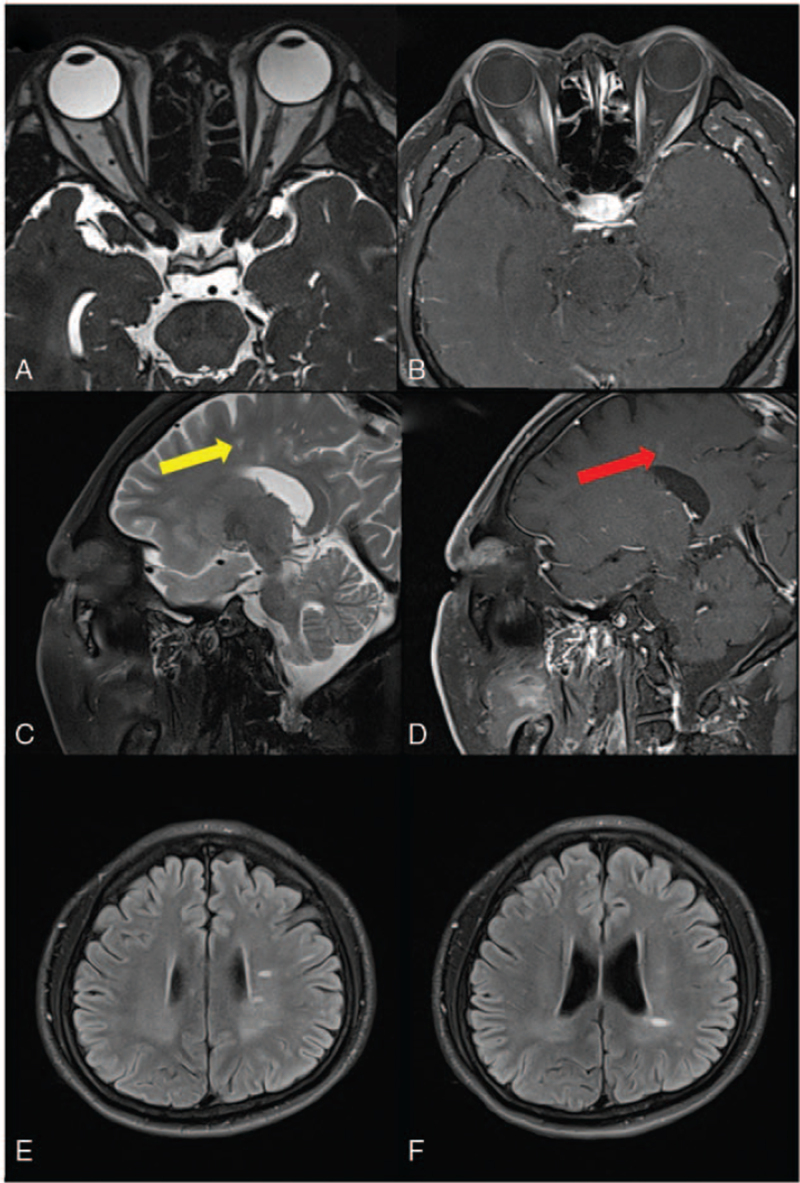
Optic nerve and brain magnetic resonance image findings during the first attack. (A) Axial constructive interference in steady state T2-weighted imaging showed high-signal intensity lesion of right optic nerve. (B) Axial postgadolinium T1-weighted MRI demonstrated enhancement of the right optic nerve in the intraorbital segment. (C, D) Sagittal T2-weighted and postgadolinium T1-weighted images showed several gadolinium-enhancing (red arrow) and nonenhancing (yellow arrow) lesions. (E, F) Axial fluid-attenuated inversion recovery images showed several high-signal intensity lesions in the left periventricular region. MRI = magnetic resonance imaging.

High-signal intensity and enhancing lesions were observed on T2-weighted magnetic resonance imaging (MRI) of the orbit in the intraorbital segment of the right optic nerve (Fig. 1A and B). Brain MRI showed multiple contrast-enhancing and noncontrast-enhancing lesions in the left periventricular region (Fig. 1C–F); no lesions were observed in the cortical, juxtacortical, infratentorial, or spinal cord regions. Intravenous corticosteroid (methylprednisolone 1000 mg/d) was administered for 3 days to treat optic neuritis of the right eye, which gradually and partially improved the decreased vision. Since the patient showed symptoms of unilateral optic neuritis, other diseases such as neuromyelitis optica spectrum disease (NMOSD) were excluded based on blood tests.

The patient was assumed to have a clinically isolated syndrome so the 2017 McDonald diagnostic criteria were used to diagnose multiple sclerosis. The patient fulfilled the “dissemination in time” criterion because both contrast-enhancing and nonenhancing lesions were seen in the left periventricular region on brain MRI. However, the “dissemination in space” criterion was not fulfilled, because lesions were not observed in any other characteristic regions other than the periventricular region. Hence, the patient was monitored during the follow-up period without any additional treatment.

**Figure 2:**
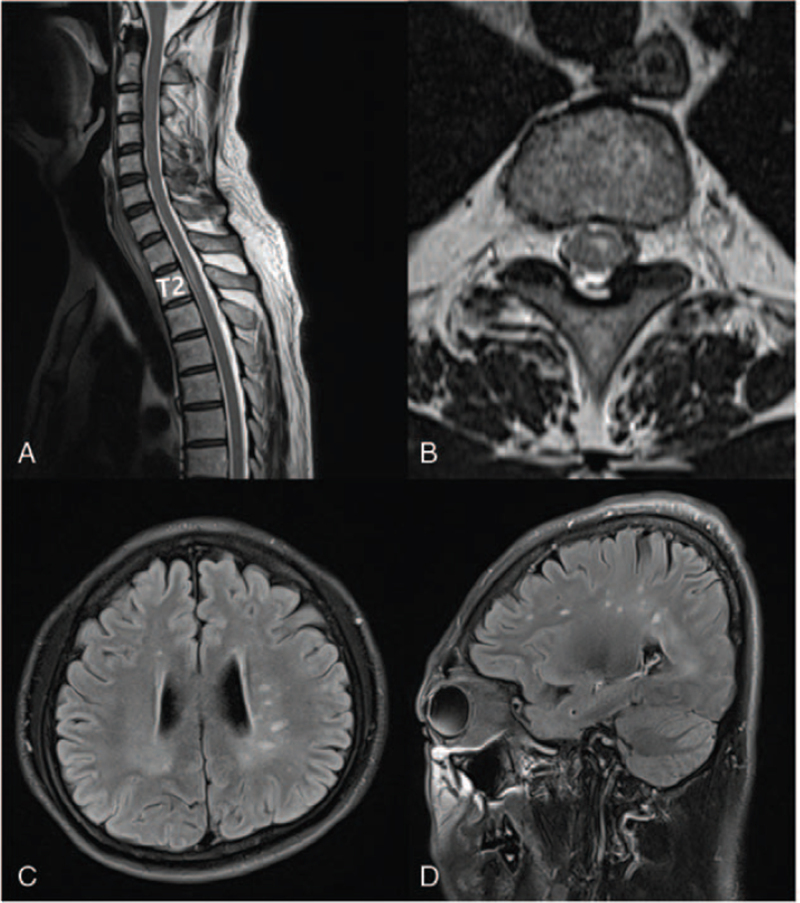
Spine and brain MRI findings during the second attack. (A, B) Sagittal and axial T2-weighted images showed intramedullary high-signal intensity lesion of the spinal cord at the level of the second thoracic vertebra. (C, D) Axial and sagittal fluid-attenuated inversion recovery images showed several high-signal intensity lesions in the periventricular and juxtacortical regions. The number of lesions had increased compared to the previous brain MRI findings. MRI = magnetic resonance imaging.

While he was receiving treatment for myasthenia gravis, the patient followed up in the outpatient department after 1 year and developed acute sensory numbness in his right trunk. On whole-spine MRI, high-signal intensity and contrast-enhancing lesions were observed at the second thoracic vertebral level (Fig. 2A and B). It was also observed that the number of high-signal intensity lesions in the periventricular and juxtacortical regions had increased in the follow-up T2-weighted brain MRI as compared to the findings from the past year (Fig. 2C and D). Based on these findings, the patient was diagnosed with recurrent multiple sclerosis. After administering high-dose steroids for 5 days, teriflunomide was initiated as a long-term disease-modifying therapy, and the progress was monitored (Figure S1, Supplemental Digital Content).

## 3 Discussion

This patient was diagnosed with ocular myasthenia gravis 6 years ago, and his symptoms were well controlled with pyridostigmine. He suffered from acute visual deterioration in the right eye and was administered with steroid treatment for optic neuritis in the same eye. Initially, he was not diagnosed with multiple sclerosis because, despite fulfilling the dissemination in time criterion of the 2017 McDonald diagnostic criteria, the lesions observed on brain MRI did not fulfill the dissemination in space criterion. Hence, the patient was monitored for disease progression.

After 1 year, the patient complained of sensory abnormality in the right trunk, and a whole spine MRI was conducted to investigate it. Myelitis was confirmed at the thoracic level of the spinal cord. Thus, a diagnosis of multiple sclerosis was made, and treatment was initiated accordingly. Strict diagnostic criteria should be implemented when diagnosing multiple sclerosis to avoid misdiagnosis, which is reportedly 25% in the United States.^[4]^ Thus, this case highlights the need for conducting shorter-interval monitoring and symptom screening in patients with autoimmune diseases, such as myasthenia gravis, considering that they have a higher probability of developing other autoimmune diseases than the general population.

Autoimmune diseases can occur with other autoimmune diseases, and 25% of the patients with autoimmune diseases tend to develop additional autoimmune diseases.^[2]^ Myasthenia gravis and multiple sclerosis are autoimmune diseases affecting the neuromuscular junctions and the CNS, respectively. Myasthenia gravis is caused by the destruction of neuromuscular junctions by an acetylcholine receptor-specific antibody, whereas multiple sclerosis is caused by neuronal antigen-specific T-lymphocytes. There are many studies and case reports on the co-occurrence of CNS demyelinating diseases and myasthenia gravis. The most recent results of these studies showed that 0.34% of patients with multiple sclerosis and 5% of patients with NMOSD were also diagnosed with myasthenia gravis, which is higher than the prevalence of myasthenia gravis (0.024%) in the general population.^[5]^

Myasthenia gravis and NMOSD are caused by the actions of the anti-acetylcholine receptor antibody and the anti-aquaporin 4 antibody, respectively. Since both diseases are mediated by the action of immunoglobulin G1 antibodies against distinct proteins, it can be speculated that a common immune mechanism is involved in both diseases.^[5]^ In contrast, since multiple sclerosis is not medicated by a specific antibody, it has fewer immune mechanisms in common with myasthenia gravis than NMOSD. Consequently, the co-occurrence rate of multiple sclerosis and myasthenia gravis may be lower than that of NMOSD and myasthenia gravis. When optic neuritis occurred in this patient, the possibility of NMOSD was higher than that of multiple sclerosis. However, the investigation revealed a diagnosis of multiple sclerosis. When optic neuritis occurs in a patient with myasthenia gravis, it is necessary to examine closely whether the diagnostic criteria for multiple sclerosis are satisfied.

When thymectomy is performed for treating myasthenia gravis, the incidence of CNS demyelinating diseases such as multiple sclerosis tends to increase.^[6]^ In the case of experimental euthymic mice that fed myelin basic protein, the onset of autoimmune encephalomyelitis was inhibited owing to oral tolerance. However, the thymectomized mice showed that oral tolerance was not achieved because absence of deletion of autoreactive T cells.^[7]^ In this case, the patient did not have severe myasthenia gravis symptoms; hence, only symptomatic treatment with pyridostigmine was administered. Immunosuppressants were not administered, and thymectomy was not performed. Nevertheless, he developed multiple sclerosis, which differentiates this case report from previous ones.

Both myasthenia gravis and multiple sclerosis result from loss of self-tolerance due to the quantitative and functional defects of T regulatory cells, which are important in immune tolerance. Moreover, they share some common immunopathological mechanisms.^[1]^ A study conducted in British Columbia^[8]^ reported that 3 out of 8 patients with co-occurring myasthenia gravis and multiple sclerosis developed multiple sclerosis approximately 6 to 8 years after the onset of myasthenia gravis. Moreover, the first manifestation of myasthenia gravis in these cases was ocular symptoms, such as ptosis or diplopia. Similarly, in this case, myasthenia gravis developed approximately 6 years prior, with ptosis and diplopia as the initial symptoms. In the British Colombia study, 5 out of the 8 patients developed optic neuritis as the first symptom of multiple sclerosis, whereas facial palsy, paresthesia, and motor weakness were the first symptoms in each of the remaining patients. It is noteworthy that optic neuritis was the first symptom of multiple sclerosis in the aforementioned study, similar to the patient in this case report. However, when the patient developed optic neuritis, his symptoms did not meet McDonald diagnostic criteria for multiple sclerosis. Therefore, follow-up observation was the only option. Hence, multiple sclerosis was diagnosed only after the occurrence of myelitis after one year, which delayed the treatment of multiple sclerosis.

## 4 Conclusion

It is extremely important to diagnose multiple sclerosis accurately and as early as possible so that prompt treatment can be given. As multiple sclerosis progresses, the severity of axonal damage increases, and the disease load accumulates even in the absence of clinical symptoms. However, as shown in this case, even if multiple sclerosis is suspected, the diagnosis may be delayed and appropriate treatment may not be initiated because the diagnostic criteria are not satisfied. Therefore, when optic neuritis occurs in patients with a neurological autoimmune disease, such as myasthenia gravis, it should always be considered as the first symptom of CNS demyelinating diseases such as multiple sclerosis rather than idiopathic optic neuritis. Therefore, shorter-interval monitoring and symptom screening should be conducted in patients with myasthenia gravis to achieve early detection of multiple sclerosis. Furthermore, additional studies are needed to determine whether separate diagnostic criteria for multiple sclerosis should be established for patients with neurological autoimmune diseases, because it is highly likely that these patients also develop other autoimmune diseases.

## Author contributions

JBB and HGK participated in the study design. JBB collected and analyzed raw clinical data. JBB and HGK performed the computational studies and wrote the manuscript. All authors have read and approved the final manuscript.

**Conceptualization:** Jeong Bin Bong, Hyun Goo Kang.

**Formal analysis:** Jeong Bin Bong, Min A Lee, Hyun Goo Kang.

**Investigation:** Min A Lee, Hyun Goo Kang.

**Methodology:** Jeong Bin Bong, Min A Lee.

**Software:** Min A Lee, Hyun Goo Kang.

**Supervision:** Hyun Goo Kang.

**Validation:** Jeong Bin Bong.

**Visualization:** Min A Lee.

**Writing – original draft:** Jeong Bin Bong.

**Writing – review & editing:** Hyun Goo Kang.

## Supplementary Material

Supplemental Digital Content
