# Supplementary figures and images for: Newly diagnosed multiple sclerosis in a patient with ocular myasthenia gravis: A case report
Source: Medicine (Baltimore). 2022 Feb 25;101(8):e28887. doi: 10.1097/MD.0000000000028887 (PMC8878705; doi:10.1097/MD.0000000000028887)

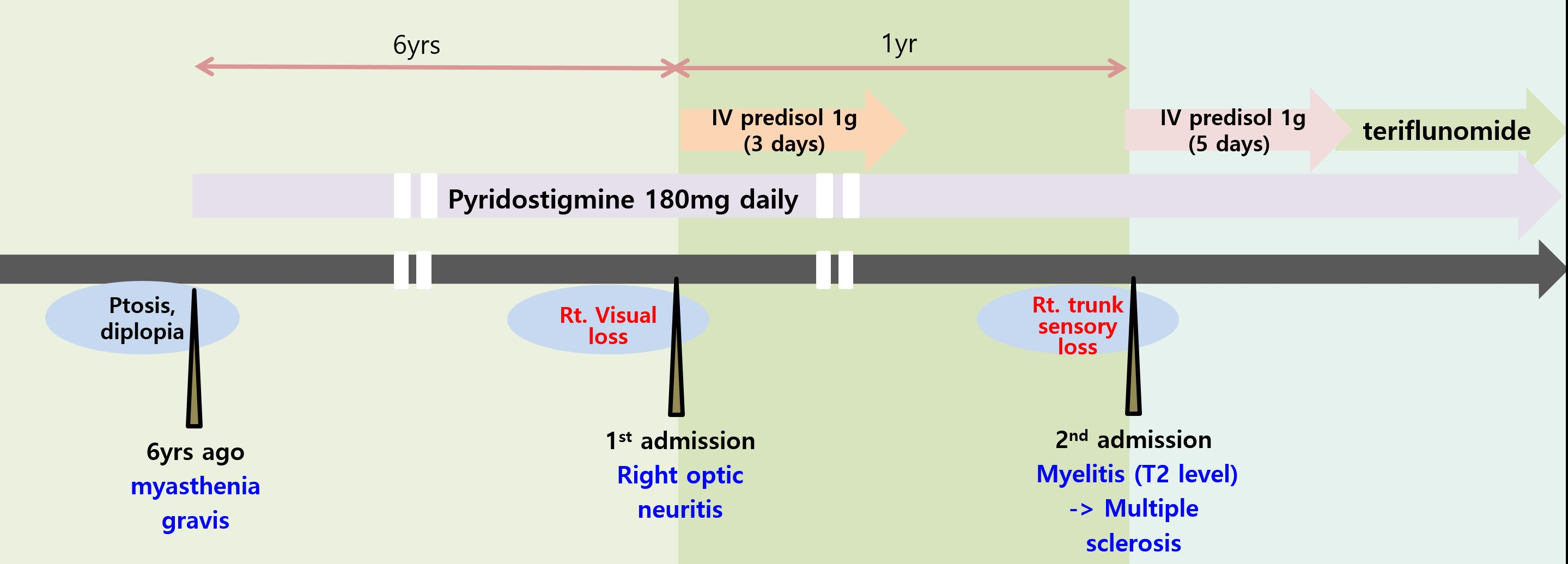

Supplement: Supplemental Digital Content [file medi-101-e28887-s001.tif]
